# Efficacy of Topical Treatments in the Management of Mild-to-Moderate Acne Vulgaris: A Systematic Review

**DOI:** 10.7759/cureus.57909

**Published:** 2024-04-09

**Authors:** Abdulaziz Althwanay, Esraa M AlEdani, Harleen Kaur, Malik Kasapoglu, Rajesh Yadavalli, Sarosh Nawaz, Tuheen Sankar Nath

**Affiliations:** 1 Dermatology, California Institute of Behavioral Neurosciences & Psychology, Fairfield, USA; 2 Dermatology, Imam Abdulrahman Bin Faisal University, Dammam, SAU; 3 Internal Medicine, California Institute of Behavioral Neurosciences & Psychology, Fairfield, USA; 4 Internal Medicine, Maharishi Markandeshwar Institute of Medical Sciences and Research, Ambala, IND; 5 Internal Medicine, Rajiv Gandhi Institute of Medical Sciences, Adilabad, IND; 6 Psychiatry, California Institute of Behavioral Neurosciences & Psychology, Fairfield, USA; 7 Surgical Oncology, Tata Medical Centre, Kolkata, IND

**Keywords:** acne management, inflammatory lesions, mild to moderate acne, acne vulgaris, topical treatments

## Abstract

Acne vulgaris, commonly called acne, is a skin condition affecting many individuals globally. It is a chronic condition characterized by developing pimples, blackheads (open comedones), whiteheads (closed comedones), and other skin lesions. Acne usually appears on the face, neck, chest, and back. It is commonly associated with puberty and adolescence but can also affect adults of all ages. Acne can be very frustrating and embarrassing, leading to low self-esteem and social isolation. The condition arises from various factors, including clogged pores, excessive sebum production, bacteria, and inflammation. This systematic review assesses the effectiveness of topical antibiotics, retinoids, niacinamide, azelaic acid, and clascoterone in treating mild-to-moderate acne vulgaris. A comprehensive search across PubMed, PubMed Central, and Google Scholar yielded 10 articles focused on topical antibiotics, with findings from 198 subjects indicating the efficacy of doxycycline against inflammatory lesions. Retinoids, such as tretinoin and adapalene, significantly improved both lesion types (open and closed comedones). Niacinamide, examined in a randomized controlled trial involving 41 participants, reduced sebum production. Another study with 60 patients revealed that azelaic acid effectively reduced both inflammatory and non-inflammatory lesions. Clascoterone emerged as a promising antiandrogenic treatment, supported by a randomized controlled trial involving 4,440 patients. It is essential that individualized therapy, incorporating patient preferences and considering adverse effects, is emphasized for optimizing acne management.

## Introduction and background

Acne vulgaris, characterized by the obstruction and inflammation of pilosebaceous units, results in various lesions such as comedones, papules, pustules, nodules, and cysts [1]. Affecting individuals across all age groups, adolescents and young adults are particularly susceptible to this dermatological condition [2]. Beyond its physical manifestations, acne significantly impacts patients' self-esteem, emotional well-being, and overall quality of life [2]. Therefore, exploring effective treatment strategies is imperative, particularly for mild-to-moderate cases.

Mild-to-moderate acne encompasses a spectrum of severity marked by comedones, papules, and pustules without extensive cystic lesions or widespread inflammation [3]. The significance of this classification lies in the variability of treatment approaches based on the condition's severity. For these cases, topical therapies often serve as the primary choice, providing localized and targeted interventions [3].

The rationale for assessing the efficacy of topical treatments for mild-to-moderate acne emanates from the need to offer practical, safe, and convenient interventions that mitigate the physical and psychological burden associated with acne. Topical therapies present several advantages, including direct application to affected areas, reduced systemic exposure, and potentially fewer adverse effects compared to systemic therapies [3]. Furthermore, evaluating the efficacy of these treatments contributes to evidence-based decision-making for dermatologists, guiding the formulation of personalized treatment plans for patients.

This systematic review aims to comprehensively evaluate the efficacy of various topical treatments for mild-to-moderate acne, including topical retinoids, topical antibiotics, combination therapies, and alternative natural treatments [4,5]. By synthesizing evidence from relevant studies, the review intends to provide valuable insights into the clinical outcomes achieved with different topical agents and their comparative effectiveness [4-6]. Additionally, the review seeks to examine the impact of these treatments on patient-reported outcomes, considering factors beyond clinical efficacy [4-6]. A rigorous methodology involving data extraction, quality assessment, and analysis enhances the review's credibility. Ultimately, this systematic review aims to advance the understanding of acne management, providing valuable guidance to healthcare providers for informed treatment decisions [4,6].

## Review

Methods

After following and applying the Preferred Reporting Items for Systematic Reviews and Meta-Analyses (PRISMA) guidelines, this review was written using the following methods [7].

Research Objective

This systematic review analyzed randomized controlled trials (RCTs) and review articles to assess the effectiveness of topical treatments in managing mild-to-moderate acne vulgaris. It sought to precisely evaluate these treatments for reducing acne symptoms, including lesion counts, patient-reported outcomes, and safety. The goal was to offer valuable insights to clinicians, researchers, and healthcare practitioners for evidence-based decision-making and to identify potential research gaps in acne management.

Inclusion Criteria

The inclusion criteria include studies involving patients of all ages diagnosed with mild-to-moderate acne vulgaris. The research specifically focused on topical treatments for this condition, considering RCTs, quasi-experimental studies, prospective and retrospective cohort studies, and review articles. Studies reported changes in acne lesion count, reductions in acne severity scores (e.g., Global Acne Assessment Scale), improvements in patient-reported outcomes, and adverse effects related to the topical treatments. The review included only English-language publications to minimize language constraints. Both published and unpublished studies, including conference abstracts and full papers, were considered to reduce publication bias. There was no restriction on the duration of the studies, allowing for a comprehensive evaluation of treatment efficacy and safety over varying time frames.

Exclusion Criteria

The exclusion criteria exclude studies involving participants not diagnosed with acne vulgaris. Additionally, these criteria exclude cross-sectional studies, case reports, case series, and expert opinions due to their limited ability to provide robust evidence. Studies without relevant outcome measures, such as those solely focusing on cosmetic outcomes without assessing acne severity, are also excluded. Only studies published in English are included, as other languages may not be feasible for comprehensive analysis. In cases of multiple publications from the same study, only the most comprehensive or recent versions are included to avoid data duplication. Finally, the criteria exclude research conducted on animal models, as it may not directly represent the efficacy and safety of treatments in humans.

Search Strategy

We conducted a comprehensive literature search using the following electronic databases: PubMed, PubMed Central, and Google Scholar. The search terms used were a combination of keywords and MeSH vocabulary terms related to Acne treatment OR (“Acne Vulgaris/drug therapy” [Majr] OR “Acne Vulgaris/therapy” [Majr]) Topical agents, Topical antibiotics OR (“Acne Vulgaris/drug therapy” [Majr] OR “Acne Vulgaris/therapy” [Majr] ), and study design (e.g., “Topical creams for acne,” pimples,” “teenage acne,” “randomized controlled trial”).

Results

Study Selection

**Figure 1:**
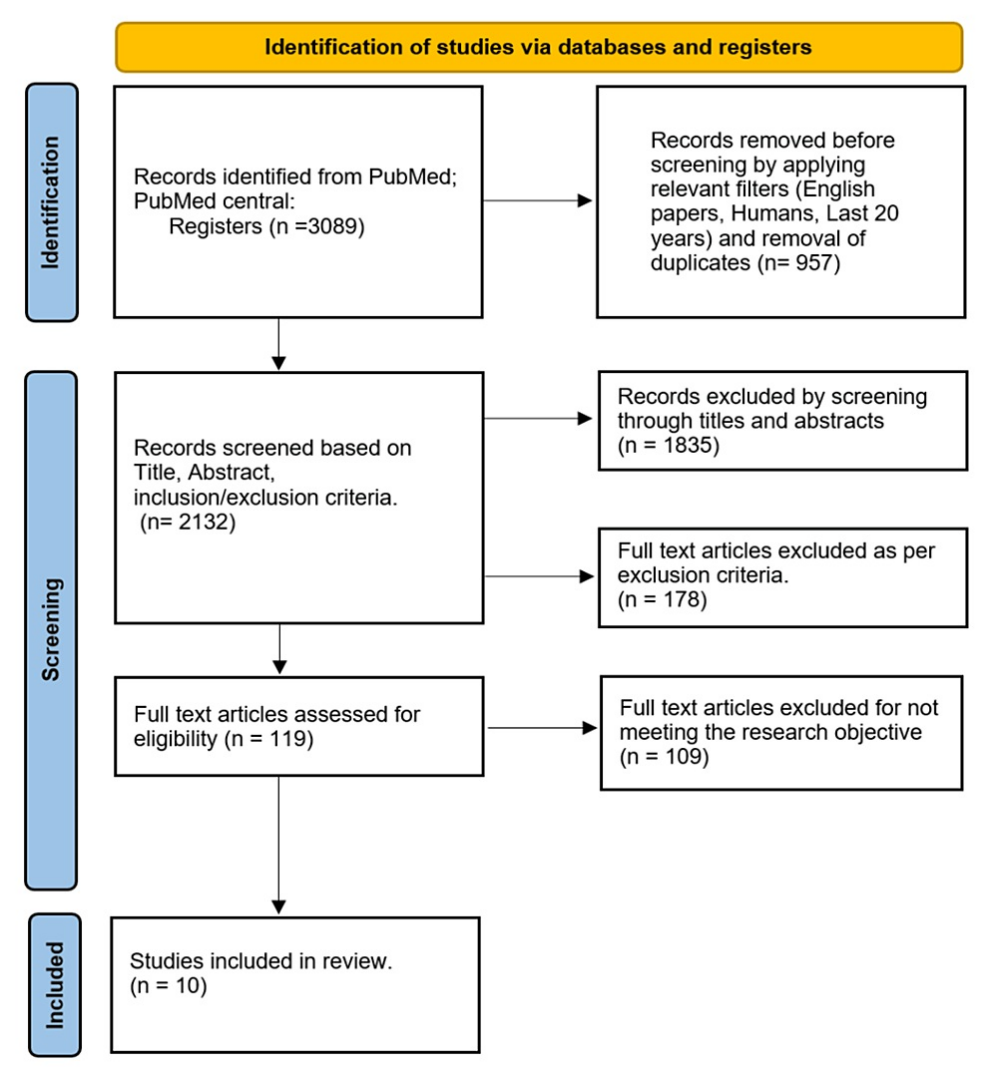
PRISMA flow diagram PRISMA, Preferred Reporting Items for Systematic Reviews and Meta-Analyses

After conducting a comprehensive and meticulous examination of studies related to this topic, we identified 10 full-text articles from a pool of 3,089 papers. We applied pertinent filters (English language, human studies, papers from the last 20 years, duplicates), excluding 957 papers. Additionally, 1,835 articles were excluded based on screening titles and abstracts. In contrast, we excluded 178 articles for not meeting the exclusion criteria. Subsequently, we eliminated 109 papers for not meeting the research objective, resulting in a final selection of 10 papers. This set of papers comprised six review articles and four RCTs. These studies involved patients of all ages diagnosed with mild-to-moderate acne vulgaris, as shown in the PRISMA flow diagram (Figure 1).

Data Extraction

Data extraction included study characteristics (authors, publication year, study design), participant characteristics (age, gender, diagnosis), intervention details (type of topical agent, duration, frequency), comparator information, and outcome data (mean change in acne severity, improvement in symptoms).

Quality Assessment

The 10 studies included (four RCTs and six review articles) were assessed for quality using the Scale for the Assessment of Narrative Review Articles (SANRA) checklist for review articles and the Cochrane Risk of Bias Assessment tool for the RCTs. Tables 1, 2 list the results of the quality assessments applied to the selected articles.

**Table 1:** Review articles assessed using the SANRA checklist on a scale of 0-2 0: Low standard (the study did not include the criterion) 1: Intermediate standard (the study included part of the criterion) 2: High standard (the study included the criterion) SANRA, Scale for the Assessment of Narrative Review Articles

	Leyden et al., 2017 [8]	Sevimli Dikicier, 2019 [9]	Tan et al., 2018 [10]	Kraft and Freiman, 2011 [11]	Kardeh et al., 2019 [12]	Piszczatoski and Powell, 2021 [13]
Justification of the article’s importance was provided	2	2	2	2	0	2
Concrete aims or forming of questions were established	2	2	2	2	2	1
The literature search was described	2	2	1	0	2	1
Referencing was done	2	2	2	2	2	2
Evidence-based reasoning was used	2	1	2	2	2	2
Appropriate presentation of information was ensured	2	1	2	2	2	2

**Table 2:** Randomized control trials (Cochrane Risk-of-Bias assessment tool) + (Positive): The study included the criterion - (Negative): The study did not include the criterion

	Ozolins et al., 2005 [14]	Del Rosso et al., 2022 [15]	Iraji et al., 2007 [16]	Saraçoğlu et al., 2011 [17]
Adequately generated allocation sequence was ensured	+	+	+	+
Adequately concealed allocation sequence was ensured	+	+	-	+
Allocation was concealed adequately	+	+	-	+
Participants and staff were blinded to the intervention	+	-	+	-
Outcome evaluators are blinded to the intervention	+	+	-	-
Incomplete outcome data were adequately addressed	+	-	+	+
Reports of the study were free from the suggestion of selective outcome reporting	+	+	+	+
The study was free of other problems that could put it at risk of bias.	-	-	-	+

Topical Retinoids

Leyden et al. published a review article that explains why topical retinoids are the centerpiece of acne treatment. Dermatology experts recommended a combination of topical retinoids and antimicrobial therapy for acne patients. Retinoids prevent the formation of comedones, resolve precursor microcomedone lesions, and exhibit anti-inflammatory properties, making them fundamental in treating acne. Recent research showed dermatologists prescribe retinoids more often (58.8%) than non-dermatologists (32.4%). The article discusses retinoids’ mechanisms of action, their role in acne management, and the evidence-based guidelines for acne treatment from the American Academy of Dermatology (AAD) and the European Dermatology Forum (EDF). The article also highlights the underuse of topical retinoids in acne treatment. [8]

Sevimli Dikicier’s study on topical treatment of acne vulgaris revealed that discontinuation rates were highest among patients using retinoids (40%), with side effects being the primary reason for discontinuation (50%). Patients with comedonal-type acne had a significantly higher rate of side effects compared to other acne types. Retinoid-containing topical medications were more frequently prescribed for patients with comedone-dominant and mixed acne types. Interestingly, every-other-night users reported the fewest side effects and lowest discontinuation rates, indicating a potential benefit of this dosing regimen. Strengths of the study on topical treatment of acne vulgaris include its focus on evaluating the efficiency, side effects, and adherence rate of current topical treatments in a sizable sample of 250 patients. The study provides valuable insights into the reasons for discontinuing therapy, particularly with retinoids, and highlights the importance of addressing side effects and treatment response in acne management. Additionally, the study’s findings on dosing regimens, such as every-other-night use, offer practical implications for optimizing treatment outcomes. However, limitations of the study may include potential biases associated with self-reported data on adherence and side effects, as well as the lack of long-term follow-up to assess treatment outcomes over time. Further research with more extended observation periods and objective measures of adherence and efficacy could strengthen the study’s findings and recommendations [9].

Tan et al. wrote that topical retinoid medications were commonly prescribed for females with mild-to-moderate acne, especially when it was predominantly comedonal. These treatments were often the first choice and were crucial for maintaining clear skin after stopping oral therapy. The recommended dosage involved applying a thin layer once daily. Three main topical retinoid medications were used: tretinoin (available in cream, gel, or microsphere gel vehicles at concentrations of 0.025-0.1%), adapalene (in 0.1% cream, gel, or lotion, and 0.3% gel), and tazarotene (in 0.05%, 0.1% cream, gel, or foam formulations). The article's strengths lie in its evidence-based recommendations supported by references to multiple studies and guidelines, providing healthcare providers with valuable information for decision-making. The emphasis on antibiotic stewardship and practical guidance for treatment administration, especially in special populations such as pregnant and lactating women, are notable strengths. However, limitations include a potential lack of novelty in presenting groundbreaking information, a focused scope that may not cover emerging therapies extensively, and the possibility of bias in selecting and interpreting referenced studies. [10].

Topical Antibiotics

Kraft and Freiman discuss the array of acne treatment options available, such as topical retinoids, benzoyl peroxide, topical and systemic antibiotics, hormonal therapies, and isotretinoin. The article offers guidance on selecting the most suitable treatment based on acne severity, type, patient’s age, gender, and medical history. For instance, topical retinoids are recommended as the first choice for mild-to-moderate acne, while systemic antibiotics are reserved for severe cases or when topical treatments prove ineffective. The article emphasizes their generally good tolerance and ability, demonstrated in numerous studies, to reduce inflammatory lesions by 46% to 70%. However, the routine use of topical antibiotics alone is discouraged due to potential resistance, which may develop within a month of daily treatment. Some argue that this resistance is irrelevant since antibiotics such as clindamycin, tetracyclines, and erythromycin inherently possess anti-inflammatory and antimicrobial properties. Yet, the risk of antibiotic-resistant strains of Staphylococcus epidermidis and Staphylococcus aureus exists with monotherapy. Combining a topical antibiotic with benzoyl peroxide helps prevent resistance. Combination therapy, such as retinoids and antibiotics together, is more effective than either agent alone. However, these agents should be applied separately unless their compatibility is established [11].

Kardeh et al. examine the effectiveness of oral azithromycin in managing mild-to-moderate acne vulgaris. Azithromycin achieves this by hindering bacterial protein synthesis. It attaches to the 50S subunit of the bacterial ribosome, preventing the movement of peptidyl-tRNA from the acceptor site to the donor site, thereby halting the elongation of the peptide chain. Consequently, this impedes bacterial growth and replication. The article scrutinized numerous clinical studies on azithromycin’s use in acne vulgaris treatment. These studies primarily delved into the appropriate dosages, treatment durations, and effectiveness of oral azithromycin compared to other antibiotic options. These studies were conducted in diverse countries and varied in methodology. Notably, one study indicated that doxycycline was a superior choice for acne treatment, showing a significant difference from azithromycin. The article thoroughly overviews current clinical studies involving azithromycin for acne vulgaris treatment. It delves into the drug’s tolerability and efficacy, comparing it with alternative antibiotic options [12].

Ozolins et al. published an RCT that was conducted to evaluate the efficacy of multiple topical antibiotics and their combinations. The study found that the percentage of participants with at least moderate improvement was 53.8% for minocycline (the least effective) and 66.1% for the combined erythromycin/benzoyl peroxide formulation (the most effective), with an adjusted odds ratio of 1.74. Benzoyl peroxide was the most cost-effective regimen, while minocycline was the least cost-effective. Oxytetracycline showed similar efficacy to minocycline but at a significantly lower cost. Reductions in acne severity were most prominent in the first six weeks of treatment, with the most significant reductions in disability scores seen with topical erythromycin-containing regimens and minocycline. The study also noted significant reductions in the prevalence and population density of cutaneous propionibacteria with topical erythromycin-containing regimens. Notably, the study did not find a net increase in the prevalence of antibiotic-resistant propionibacteria with any regimen, and most participants lost resistant strains during treatment. The findings suggest that topical antimicrobial therapy can be as effective as oral antibiotics for managing mild-to-moderate inflammatory acne of the face, with an early review at six weeks recommended for treatment adjustment if needed. The study’s strengths included its rigorous randomized controlled design, intention-to-treat analysis, and the inclusion of multiple treatment regimens, providing a comprehensive comparison of different therapeutic approaches. The study also collected bacterial resistance data before and after treatment, shedding light on the response variation.

Additionally, the study was industry-independent, reducing potential bias, and employed assessors who were not dermatologists, minimizing preconceived notions about treatment efficacy. However, the study also has limitations, such as a higher-than-anticipated dropout rate of 27%, potentially affecting the generalizability of the results. The study’s focus on facial acne limits its external validity, and the requirement for participants to stop all active acne treatment for a four-week washout period may have influenced the willingness of individuals to participate. These limitations should be considered when interpreting and applying the study’s findings to clinical practice [14].

Del Rosso et al. published a 12-week multicenter study, initiated on July 28, 2020, and completed on April 30, 2021, employed a double-blinded design, comparing once-daily trifarotene cream 50μg/g plus enteric-coated doxycycline 120mg (T+D) against trifarotene vehicle plus doxycycline placebo (V+P) with a 2:1 randomization among 198 subjects aged 12 and older with facial acne (Investigator’s Global Assessment [IGA] score of 4). Inclusion criteria encompassed an IGA score of 4, age ≥12 years, and a total lesion count of at least 20 inflammatory and non-inflammatory lesions, emphasizing compliance with study requirements. Excluded were patients with a history of hypersensitivity to tetracycline antibiotics, pregnant or breastfeeding women, and individuals with inflammatory bowel disease, photosensitivity, or other skin conditions potentially affecting acne evaluation. The protocol, aligned with the Declaration of Helsinki and the International Conference on Harmonization/Good Clinical Practice, was approved by the Western Institutional Review Board (WIRB). A key amendment allowed the enrollment of subjects with up to four nodules or cysts (combined) and clarified total lesion counts, addressing mask-related effects. Strengths included a randomized, placebo-controlled design, multicenter implementation, a sizable sample size, and incorporation of diverse subgroups, enhancing generalizability. Validated outcome measures like IGA score and total lesion count bolstered result reliability. However, limitations encompassed the study’s 12-week duration, potential lack of blinding for doxycycline, exclusion of specific patient groups, anticipation of dropouts and non-evaluable subjects, and limited insight into the treatment’s long-term safety and adverse effects [15].

Topical Clascoterone

Piszczatoski and Powell explored topical clascoterone and how it operates by inhibiting androgen receptors in the skin, thereby reducing sebum production and inflammation, leading to fewer acne lesions. Acne vulgaris results from excess sebum production, inflammation, and bacterial overgrowth, with androgens, particularly testosterone, playing a pivotal role in acne development by stimulating sebum production and inflammation. The cream is applied topically twice daily for 12 weeks on affected areas. The outcomes of two phase 3 clinical trials demonstrated that clascoterone cream, 1%, was significantly more effective than the control cream in achieving treatment success. Success was defined as an IGA score indicating clear or almost clear skin and at least a two-grade reduction from baseline by week 12.

Additionally, the cream was well-tolerated, with no severe adverse events reported. The study concluded that clascoterone cream, 1%, offers a safe and effective treatment for acne vulgaris with a favorable safety profile. This innovative approach brings hope to those dealing with acne. The study’s strengths lie in its randomized, double-blinded, vehicle-controlled design across two identical phase 3 clinical trials, ensuring robust and reliable results. The large sample size of 1,440 participants enhances statistical power. At the same time, using established measures such as the IGA score and total lesion count strengthens the study’s validity. The study also reported the cream’s safety with no serious adverse effects. However, limitations include the study’s focus solely on a specific group with moderate-to-severe facial acne. This limits the broader application of results, especially to individuals with milder or non-facial acne. The absence of a long-term follow-up period hinders understanding the treatment’s durability beyond 12 weeks. Furthermore, the study did not compare clascoterone cream, 1%, with other common acne treatments such as benzoyl peroxide or topical retinoids, restricting comparisons of efficacy and safety. Lastly, the study’s funding source from the clascoterone cream manufacturer raises potential bias concerns in result interpretation [13].

Topical Azelaic Acid

Iraji et al.’s study involved 60 patients with mild-to-moderate acne vulgaris, randomly divided into two groups receiving topical azelaic acid gel and a placebo, respectively. Inclusion criteria comprised patients aged 15-40 years with mild-to-moderate acne. In contrast, exclusion criteria included severe acne, pregnancy or lactation, recent systemic or topical acne treatments, and use of other topical medications during the study. Over 45 days, patients were monitored every 15 days, evaluating treatment response through total acne lesions counting (TLC) and acne severity index (ASI), analyzed statistically using t-tests and Statistical Package for Social Sciences (SPSS; IBM Corp., Armonk, NY). The study employed a randomized, double-blinded design, which minimized bias, enhancing reliability. Strengths included the gold standard of a randomized, double-blinded clinical trial, meticulous inclusion/exclusion criteria, comprehensive monitoring, and objective measures. Similar and tolerable side effects suggested topical azelaic acid gel’s suitability for mild-to-moderate acne vulgaris. However, limitations included a small sample size, potentially restricting generalizability, and a relatively short study duration, possibly insufficient to assess long-term efficacy and safety. Additionally, the study did not explore azelaic acid’s mechanism of action, limiting understanding. Careful consideration of study design and execution is crucial to ensuring reliable results applicable to a broader population, guarding against biases such as sample size limitations, lack of blinding, and the absence of objective measures for evaluating treatment response [16].

Topical Niacinamide

Topical niacinamide has proven effective in treating acne vulgaris due to its diverse mechanisms of action. One of its primary functions is its anti-inflammatory effect. Niacinamide has demonstrated significant anti-inflammatory properties, impacting neutrophil chemotaxis, histamine release inhibition, suppression of the lymphocyte transformation test, and cytokine-induced reduction of nitric oxide synthase in various cells. These actions decrease inflammation, a pivotal factor in acne lesion development. Besides its anti-inflammatory attributes, niacinamide offers additional benefits to the skin. It enhances protein and keratin synthesis, stimulates ceramide production, and accelerates keratinocyte differentiation. These effects stabilize the epidermal barrier and improve the skin’s moisture content, promoting overall skin health and appearance while potentially preventing new acne lesions.

Moreover, niacinamide has exhibited inhibitory effects on photocarcinogenesis, the process through which ultraviolet radiation can cause skin cancer. This discovery suggests its potential use in individuals at high risk of skin cancer or with a history of the disease. However, the study had limitations. The findings may lack broad applicability with a small sample of 41 patients. Additionally, it focused only on mild-to-moderate acne, leaving uncertainty about its effectiveness for severe cases. The lack of a control group makes it challenging to attribute improvements solely to niacinamide, as other factors such as the natural progression of the disease or the placebo effect could be at play. Reliance on self-reported treatment satisfaction introduces potential bias, possibly not accurately reflecting the treatment’s effectiveness. Furthermore, long-term safety and efficacy and the treatment’s cost-effectiveness remain unknown, raising important considerations for patients and healthcare providers when choosing a treatment approach [17].

Discussion

This systematic review has evaluated the diverse landscape of topical agents in pursuing effective solutions for mild-to-moderate acne vulgaris. Acne, a prevalent dermatological condition, necessitates nuanced and evidence-based approaches to alleviate its physical and psychological impact.

Topical Agents With Bacteriostatic and Bactericidal Effects

We critically selected the studies conducted by Iraji et al., Kraft and Freiman, Kardeh et al., Ozolins et al., and Del Rosso et al. to deepen our understanding of the bactericidal and bacteriostatic effects of topical agents in the treatment of mild-to-moderate acne vulgaris. Iraji et al.’s research provides valuable insights into how topical azelaic acid combats acne-causing bacteria, shedding light on their bactericidal properties. Similarly, the studies by Del Rosso et al., Ozolins et al., and Kardeh et al. delve into the broader bacteriostatic effects, emphasizing the ability of topical treatments to inhibit bacterial growth, thus preventing the exacerbation of acne lesions. The findings of Kraft and Freiman contribute to this discussion by exploring the comparative efficacy of different topical agents in achieving bactericidal and bacteriostatic outcomes.

Topical azelaic acid: Topical azelaic acid emerged as a promising solution in dermatology for combating acne vulgaris. This common skin condition affected millions worldwide. This naturally occurring dicarboxylic acid, derived from grains such as barley, wheat, and rye, showcased remarkable efficacy in treating various forms of acne. Its mechanism of action primarily involved inhibiting the growth of acne-causing bacteria and preventing the accumulation of keratinocytes in hair follicles, which often led to acne lesions. Unlike many other acne treatments, azelaic acid’s anti-inflammatory properties reduce redness and swelling associated with acne breakouts, making it a comprehensive and versatile therapeutic option. As dermatological research unraveled its potential, topical azelaic acid stood at the forefront of innovative strategies, offering individuals suffering from acne a renewed sense of confidence and skin health. [16].

Iraji et al. conducted a study to assess the effectiveness of topical azelaic acid gel in treating mild-to-moderate acne vulgaris. This double-blinded, randomized clinical trial involved 60 participants randomly divided into two groups: one receiving 20% azelaic acid gel and the other receiving a placebo gel. The findings indicated that azelaic acid gel was significantly more effective than the placebo in reducing TLC and improving acne severity. Specifically, azelaic acid gel reduced the mean TLC by 60.6% compared to a 19.9% reduction with the placebo (P = 0.002). The ASI was decreased by 65.2% with azelaic acid gel compared to a 21.3% reduction with the placebo (P = 0.001). This meant that azelaic acid gel was 3.04 times more effective than the placebo in reducing TLC [16].

Topical antibiotics: Regarding antibiotics, topical erythromycin and clindamycin were generally well-tolerated and effectively reduced inflammatory lesions by 46% to 70% in multiple RCTs. The primary purpose of using topical antibiotics in acne treatment was to decrease the population of Propionibacterium acnes (P. acnes) bacteria on the skin, which played a role in developing acne lesions. However, it was crucial to understand that relying solely on topical antibiotics should not have been a common practice, as P. acnes bacteria could develop resistance within a month of daily treatment initiation. Antibiotic resistance could also lead to the emergence of antibiotic-resistant strains of Staphylococcus epidermidis and Staphylococcus aureus. To counter resistance, topical antibiotics were frequently combined with other agents, such as benzoyl peroxide, which could enhance their efficacy and lower the risk of resistance [11,12].

Ozolins et al. compared multiple antimicrobial therapies for acne and yielded insightful results. The authors found that the percentage of participants with at least moderate improvement was 53.8% for minocycline (the least effective) and 66.1% for the combined erythromycin/benzoyl peroxide formulation (the most effective), with an adjusted odds ratio of 1.74. Benzoyl peroxide was the most cost-effective regimen, while minocycline was the least cost-effective. Oxytetracycline showed similar efficacy to minocycline but at a significantly lower cost. Reductions in acne severity were most prominent in the first six weeks of treatment, with the most significant reductions in disability scores seen with topical erythromycin-containing regimens and minocycline. The study also noted significant decreases in the prevalence and population density of cutaneous propionibacteria with topical erythromycin-containing regimens. Notably, the study did not find a net increase in the prevalence of antibiotic-resistant propionibacteria with any regimen, and most participants lost resistant strains during treatment. The findings suggested that topical antimicrobial therapy could be as effective as oral antibiotics for managing mild-to-moderate inflammatory acne of the face, with an early review at six weeks recommended for treatment adjustment if needed [14].

Del Rosso et al. discussed a randomized, placebo-controlled, phase IV study that evaluated the efficacy and safety of trifarotene cream plus doxycycline compared to vehicle/placebo in patients with facial acne. The study enrolled 202 subjects aged 12 and older, with an approximate even distribution of male-to-female subjects, adults (≥18 years), and adolescent subjects (12-17 years). The study found that trifarotene cream plus doxycycline was more effective than vehicle/placebo in reducing total lesion counts. The incidence of treatment-emergent adverse events was similar between the two groups. Patients in the trifarotene cream plus doxycycline group reported more significant improvements in acne-specific quality of life and subject satisfaction/drug acceptability. The study suggested that trifarotene cream plus doxycycline may be an effective and safe treatment option for patients with mild-to-moderate facial acne [15].

Topical Agents With Anti-inflammatory and Desquamative Effects

Topical agents with anti-inflammatory and desquamative effects are widely used in dermatology to treat various skin conditions. These agents work by reducing inflammation, redness, and scaling on the skin. This systematic review delves into the pivotal work of Tan et al., Leyden et al., Sevimli Dikicier, and Saraçoğlu et al. to comprehensively analyze the impact of these topical agents on acne management. Acne, a multifactorial skin disorder, necessitates interventions that address inflammation and regulate the shedding of skin cells. Leyden et al., Tan et al., and Sevimli Dikicier’s contributions provide valuable insights into the intricate interplay between topical retinoids' anti-inflammatory and desquamative effects. Saraçoğlu et al. conducted a study on topical niacinamide, a popular ingredient in skincare products due to its ability to improve skin texture, reduce inflammation, and minimize the appearance of pores.

Topical retinoids: Topical retinoids are considered the mainstay therapy for acne because they effectively treat multiple aspects of acne’s complex pathophysiology. Retinoids work by normalizing the desquamation process in the skin, reducing keratinocyte proliferation, and promoting differentiation, which helps to prevent the formation of microcomedones. Retinoids also have anti-inflammatory properties and block several critical inflammatory pathways activated in acne, including toll-like receptors, leukocyte migration, and the activator protein 1 (AP-1) pathway. These mechanisms of action make retinoids an essential component of acne therapy, as they target both the comedonal and inflammatory components of the disease. Evidence-based guidelines for acne treatment, including those from the AAD and the EDF, recommend topical retinoids as a first-line treatment for mild-to-moderate acne. Overall, the effectiveness and safety of topical retinoids, combined with their unique mechanisms of action, make them the mainstay therapy for acne. The article mentioned above discusses the mechanisms of action of retinoids, their role in acne management, and the evidence-based guidelines for acne treatment from the AAD and the EDF [8].

According to Sevimli Dikicier’s study, patients using retinoids had the highest discontinuation rates (40%), and the most common reason for discontinuation was side effects (50%). Patients who had comedonal-type acne experienced more side effects compared to other types of acne. Retinoid-containing topical medications were prescribed more frequently for patients who had comedone-dominant and mixed acne types. Interestingly, patients who used retinoids every other night reported fewer side effects and lower discontinuation rates, which suggests that this dosing regimen might have some advantages [9].

Topical retinoid medications were commonly prescribed for females with mild-to-moderate acne, especially when it was predominantly comedonal. These treatments were often the first choice and were crucial for maintaining clear skin after stopping oral therapy. The recommended dosage involved applying a thin layer once daily. Three main topical retinoid medications were used: tretinoin (available in cream, gel, or microsphere gel vehicles at concentrations of 0.025-0.1%), adapalene (in 0.1% cream, gel, or lotion, and 0.3% gel), and tazarotene (in 0.05%, 0.1% cream, gel, or foam formulations) [10].

Topical niacinamide: Topical niacinamide represented a significant advancement in the dermatological arsenal against acne vulgaris. Niacinamide, a form of vitamin B3, has gained attention for its multifaceted benefits in skin health. Its anti-inflammatory and sebum-regulating properties made it a promising candidate for managing acne. Topical niacinamide reduces existing lesions by targeting the underlying factors contributing to acne formation. It helped prevent new breakouts, offering a gentle yet practical approach to achieving clearer and healthier skin [17-19].

Saraçoğlu et al. explored the effectiveness of a 4% niacinamide gel applied topically in treating mild and moderate acne vulgaris; this study involved 41 participants, with 33 females, and an average age of 21.6 years. Over eight weeks, patients’ acne lesions were assessed biweekly, and the reduction in lesion numbers determined the treatment’s efficacy. The results demonstrated that topical niacinamide gel was safe and significantly effective in decreasing the number of pustules, comedones, and papules in individuals with acne vulgaris. Following the treatment period, three patients expressed dissatisfaction, five reported mild satisfaction, 14 were moderately satisfied, 11 were quite satisfied, and five were very satisfied. Dermatological evaluations post-treatment revealed the treatment’s ineffectiveness in two patients, mild improvement in 10 patients, moderate improvement in 12 patients, significant improvement in nine patients, and complete resolution in five patients. The study concluded that topical niacinamide gel was a safe and effective treatment for mild and moderate acne vulgaris [17].

Topical Agents With Antiandrogenic Effects

Topical clascoterone, a novel antiandrogen medication, emerged as a groundbreaking solution for managing acne vulgaris. Unlike traditional treatments, clascoterone specifically targets androgen receptors in the skin, disrupting the hormonal pathways that contribute to acne development. By inhibiting these receptors, clascoterone helped regulate sebum production and reduce inflammation, effectively combating acne lesions [13].

Piszczatoski and Powell summarized the outcomes of two phase III RCTs, which provided crucial data to the U.S. Food and Drug Administration to approve topical clascoterone in treating acne vulgaris. These trials involved 1,440 participants, half using clascoterone 1% cream and the other half using a placebo cream. The results indicated that clascoterone was significantly more effective than the placebo in reducing both non-inflammatory and inflammatory lesions of acne vulgaris. Specifically, the group using clascoterone 1% cream twice daily for 12 weeks showed success rates of 18.4% compared to 9.0% in the placebo group. Clascoterone decreased non-inflammatory lesions by -19.4 versus -13.0 absolute lesion count and inflammatory lesions by -19.3 versus -15.5 total lesion count. The trials revealed that clascoterone was generally well-tolerated, with minimal adverse effects reported. These findings suggested that topical clascoterone was a safe and effective treatment for acne vulgaris [13].

## Conclusions

In conclusion, our systematic review delves into the topical management of mild-to-moderate acne vulgaris, evaluating the efficacy of various agents, including antibiotics, retinoids, niacinamide, azelaic acid, and clascoterone. Our analysis revealed that topical antibiotics effectively reduce inflammatory lesions, while retinoids significantly improve overall acne severity, particularly in managing non-inflammatory lesions. Niacinamide is a promising option, displaying anti-inflammatory properties and enhancing overall skin texture. With its dual antimicrobial and anti-inflammatory effects, azelaic acid proves valuable in reducing both inflammatory and non-inflammatory lesions. Additionally, clascoterone, as an androgen receptor antagonist, offers a novel approach by targeting sebaceous gland androgen receptors, effectively reducing sebum production and inflammatory lesions. Tailored, personalized approaches considering patient preferences and characteristics are vital in choosing the most suitable treatment. Acknowledging study limitations, including diverse methodologies, this review emphasizes the need for ongoing research, focusing on long-term safety, combination therapies, and innovative agents to continually refine acne management strategies for optimal patient outcomes.
